# Effects of Blood Flow Restriction Training on Lower Extremity Maximum Dynamic Strength and Isokinetic Muscle Strength among Athletes: A Systematic Review and Meta-Analysis

**DOI:** 10.5114/jhk/195726

**Published:** 2025-06-02

**Authors:** Rui Li, Chen Soon Chee, Johan bin Abdul Kahar, Tengku Fadilah Tengku Kamalden, Kun Yang, Zhendong Gao

**Affiliations:** 1 Department of Sports Studies, Faculty of Educational Studies, Universiti Putra Malaysia, Selangor, Malaysia.; 2 Department of Orthopaedic, Faculty of Medicine and Health Sciences, Universiti Putra Malaysia, Selangor, Malaysia.; 3 National Sports Institute, National Sports Complex, Kuala Lumpur, Malaysia.

**Keywords:** kaatsu training, arterial occlusion pressure, squat, peak torque, cuff width

## Abstract

This systematic review aimed to examine the effects of blood flow restriction training (BFRT) on lower extremity muscle strength of athletes. This study followed the PRISMA-P guidelines. A comprehensive search for literature up to August 2024 was conducted on Scopus, Web of Science, PubMed and EBSCOhost to determine the eligible studies based on the inclusion criteria. The Cochrane risk of bias assessment tool was employed to evaluate the methodological quality of studies, the certainty of the evidence was assessed utilizing the Grading of Recommendations, Assessment, Development, and Evaluation (GRADE), and the subgroup analyses were conducted based on moderator factors. A total of sixteen studies with 366 healthy athletes aged 15–27 years were included in the meta-analyses. The results indicated a moderate to large effect size (ES) of BFRT on the isokinetic knee flexion peak torque (ES = 0.88; p < 0.05), lower extremity dynamic strength-1RM (ES = 0.99; p < 0.001), and isokinetic knee extension peak torque (ES = 1.47; p < 0.001). The findings of subgroup analysis revealed that BFR-RT training (ES = 1.20–1.95; p < 0.05), training frequency of ≥ 3 times per week (ES = 1.13–1.16; p < 0.001), occlusion pressures of ≥ 160 mmHg (ES = 1.32–2.23; p < 0.01) and cuff width of > 7 cm (ES = 1.84–11.84; p < 0.01) were more beneficial to isokinetic muscle strength. No significant difference was observed in training duration (p > 0.05). In conclusion, blood flow restriction training is effective in enhancing lower extremity muscle strength in healthy athletes.

## Introduction

Lower extremity muscle strength (LEMS) is the force individuals can generate during the maximum contraction of specific lower limb muscle groups, forming the foundation for athletes to perform various movements (Slimani et al., 2018). Generally, methods to measure maximal strength include maximal dynamic strength testing (one repetition maximum [1RM]) and peak force or torque achieved by skeletal muscles during physical activities (Chelly et al., 2010). The ability of athletes to execute sustained high-intensity movements during competition or training reflects the correlation between LEMS and the specificity of motor skills (Young, 2006). Motor skill specificity entails meticulously designed body movements within specific environmental and temporal variables to achieve goals, incorporating muscle contraction kinetics, joint range of motion, and utilization of energy systems (Graham-Smith et al., 2012), for instance, a basic half-squat stance, rebounding for rebounds, and acceleration-deceleration sprints in basketball (Bradic et al., 2009), speed and explosiveness in track and field (Bazyler et al., 2017) and absolute strength and endurance in combat sports (Chaabene et al., 2018). Furthermore, the mechanisms of muscle strength development involve neuromuscular adaptation (Moritani, 1993), and the interaction between the neurological system and muscles is illustrated in the stretch-shortening cycle (SSC) (Nicol et al., 2006). Through repetitive strength training, the nervous system can optimize the recruitment and regulation of motor units, thereby enhancing the efficiency of neuromuscular connections (Jensen et al., 2005). Meanwhile, effective training leads to muscle fiber hypertrophy and an increased muscle cross-sectional area (CSA), prompting structural changes in muscles and facilitating greater force output (Bompa et al., 2012). Therefore, the training status of athletes is crucial for their technical performance and competitive level, thus scientific training methods are required to develop LEMS (Suchomel et al., 2018).

Resistance training (RT) is a widely adopted method for enhancing LEMS, with the training load generally at 70%–85% of the individual's 1RM (ACSM, 2009). RT has been proven to effectively induce changes in muscle function and increase muscle strength and the muscle CSA (Bryanton et al., 2012). Several studies have suggested that high-intensity knee extension exercises can increase the CSA of knee extensor and maximum dynamic strength in athletes (Faigenbaum and Myer, 2010; Schoenfeld et al., 2017). Additionally, high-intensity RT (HI-RT) activates the hamstring muscle function and improves motor performance of driving muscles (Granacher et al., 2016; Harries et al., 2012; Lesinski et al., 2016; Rosenberger et al., 2017; Skalski et al., 2024). Nevertheless, this training modality is not without drawbacks. In multi-joint training, the pathways for muscle force generation are decreased due to mechanical disadvantages at specific joint angles, which potentially results in deceleration in upward movements and a failure to effectively improve muscle strength (van den Tillaar et al., 2014). Moreover, traditional RT comes with several potential risks and challenges (Alqarni, 2019; Damas et al., 2016). Long-term strength training with high loads can exacerbate joint stress and soft tissues, which may result in chronic damage to the athletes' musculoskeletal system (Lavallee and Balam, 2010; Siewe et al., 2014). For instance, basketball players experience muscle fatigue, muscle stiffness, and decreased kinetic power after strength training, which can affect performance (Doma and Deakin, 2015). Therefore, in pursuit of safer and more effective training methods, researchers are gradually leaning towards adopting blood flow restriction training (BFRT).

BFRT applies external pressure utilizing pneumatic tourniquets or cuffs (automatic or manual) wrapped around the proximal part of the limb (Fahs et al., 2012). The vascular system beneath the cuff is gradually compressed mechanically when the cuff is inflated, leading to full restriction of venous return and partial restriction of arterial inflow, enabling low-load training under limb ischemia (Loenneke et al., 2012) also known as kaatsu training or occlusion training (Manini and Clark, 2009). Interestingly, several studies have demonstrated that BFRT at 20%–30% of 1RM can produce strength gains similar to those achieved with HI-RT at 70%–85% of 1RM (Giles, 2016; Pignanelli et al., 2021; Wang et al., 2023b; Yasuda et al., 2011). However, the mechanism of BFRT differs from HI-RT in that it does not rely on high external loads to induce muscle adaptation but instead induces metabolic stress and mechanical tension within muscle fibers (Patterson et al., 2019). Furthermore, the vascular system proximal to skeletal muscle is progressively mechanically compressed under conditions of the restricted blood flow, leading to an inadequate supply of oxygen within muscle tissue (Larkin et al., 2012). Increased metabolic stress during muscle contraction under hypoxic conditions contributes to the accumulation of lactate metabolites (de Freitas et al., 2017), mimics the metabolic environment associated with high-load exercise (Faller et al., 2023), and promotes the release of muscle growth hormone (Takarada et al., 2000), which can enhance muscle strength. On the other hand, lower loads reduce mechanical stress on joints and connective tissues compared to traditional strength training, which minimizes the risk of injury (Gabbett, 2016). Several studies have proven that BFRT is safer, of lower risk, and more efficient than HI-RT (Ferraz et al., 2018; Kelly et al., 2020; Loenneke et al., 2011). Powerful strength exercises allow athletes to better integrate their muscles and control movements (Suchomel et al., 2016). Increasingly, research has confirmed the efficacy of BFRT in improving LEMS, including the peak torque of knee extension and flexion (Martín-Hernández et al., 2013), maximum dynamic strength (Lixandrão et al., 2015), and maximal voluntary contraction of the quadriceps and hamstrings (Golubev et al., 2021). Therefore, it is reasonable to believe that BFRT has beneficial effects on LEMS in healthy athletes.

The efficacy of BFRT has become an active area of research. Existing systematic reviews and meta-analyses have consolidated the effects of BFRT on explosive power of lower limbs (Wang et al., 2023) and LEMS in older adults (Zhang et al., 2022) as well as healthy populations (Grønfeldt et al., 2020; Yang et al., 2022). Nevertheless, previous meta-analyses focused mainly on the upper limbs and did not concern lower extremity muscle strength of healthy athletes. In fact, the large muscle groups of upper limbs are located mainly proximal to the occlusive cuff pressure, whereas the large muscle groups of the lower extremity are located mainly distal (Dankel et al., 2016). Thus, training adaptations are different for both muscle groups. Additionally, the predictive effects for trained and untrained individuals appear to be different. Therefore, this study aimed to guide evidence-supported practice by systematically synthesizing published data on the effects of BFRT on LEMS in healthy athletes. We attempted to explore the impact of BFRT on athletes' lower limb maximum dynamic strength and isokinetic muscle strength through meta-analysis and evaluating the influence of moderators in BFRT to optimize training protocols.

## Methods

In this systematic review, the data collection, selection, and analysis followed the Preferred Reporting Items for Systematic Reviews and Meta-Analysis Programs (PRISMA-P) guidelines (Moher et al., 2015). Additionally, this review has been registered on Inplasy.com (INPLASY202390051).

### Search Strategy

An extensive search on this topic was conducted using electronic databases including Scopus, Web of Science, PubMed, and EBSCOhost to collect publications until August 2024 relevant to this study. The search strategy and keywords are detailed in Table 1. Additionally, Google Scholar was searched to avoid missing any potential articles that met the criteria. We also screened the reference lists for more valuable citations from the included articles and previous relevant reviews and meta-analyses.

**Table 1:** Detailed search strategy for the databases.

Database	Detailed search strategy	Results
Scopus	(TITLE-ABS-KEY (“blood flow restriction training” OR “kaatsu training” OR “occlusion training” OR “ischemia*”) AND TITLE-ABS-KEY (“lower limb muscle strength” OR “muscular function” OR “dynamic muscle strength” “isokinetic muscle strength” OR “isometric muscle strength” OR “peak torque”) AND TITLE-ABS-KEY (“athletes” OR “players” ))	10
Web of Science	(AB= ((“blood flow restriction training” OR “kaatsu training*” OR “occlusion training” OR “vascular occlusion training” OR “resistance training combined with BFR”))) AND AB= ((“lower extremity muscle strength” OR “lower limb muscle function” OR “isokinetic muscle strength” OR “peak torque” OR “dynamic muscle strength”))) AND AB= (“athletes” OR “player”))	32
PubMed	(“blood flow restriction training”[Title/Abstract] OR “kaatsu training”[Title/Abstract] OR “occlusion training”[Title/Abstract] OR “ischemia*”[Title/Abstract]) AND “lower limb muscle strength”[Title/Abstract]) OR “dynamic muscle strength”[Title/Abstract] OR “isokinetic muscle strength”[Title/Abstract] OR “isometric muscle strength”[Title/Abstract] OR “peak torque”[Title/Abstract]) AND “athletes”[Title/Abstract]) OR “players”[Title/Abstract])	1716
EBSCOhost	AB (“Blood flow restriction training” OR “kaatsu training” OR “occlusion training”) AND AB (“lower limb muscle strength” OR “muscular function” OR “isokinetic muscle strength” OR “dynamic muscle strength” OR “peak torque”) AND AB (“athletes” OR “players”)	15

### Study Selection and Data Extraction

Following the article retrieval process, all records were imported by the first author into the Endnote X9 software and duplicate entries were eliminated. Data were extracted from the included articles by two independent reviewers (R.L. and K.Y.) through a thorough examination of the full texts, with organization facilitated using Microsoft Excel records. The third reviewer (C.S.C.) reviewed the data to render the final decision. The extracted information considered the following elements: (a) authors and publication year; (b) subjects’ characteristics (including sample size, sex, age, and the type of the athlete); (c) intervention details, comprising training frequency, duration, the exercise load, occlusion pressure, the occlusion mode, and the training protocol; (d) outcome measurements. If the necessary data were not provided, the authors of the studies were contacted to acquire the missing information.

### Eligibility Criteria

According to the PICOS strategy, the eligible studies were selected based on the following inclusion criteria: (a) all levels of healthy athletes without age restrictions; (b) the use of BFRT or BFRT in combination with other training modalities; (c) comparison between the BFRT group and the control group; (d) outcome metrics related to maximum dynamic press strength of the lower extremity, isokinetic knee extension and flexion peak torque; (e) randomized controlled trial (RCT) studies; (f) studies written in English. Studies were disregarded if any of the following criteria were met: (a) athletes with sports injuries; (b) no BFRT program or BFRT combined with non-sports exercises (e.g., mental training); (c) presentation of data in graphical forms only with authors of those studies not responding regarding further clarification.

### Methodological Quality Assessment

Methodological quality in the RCT was assessed based on the Cochrane Collaboration Assessing Risk of Bias Tool and using RevMan 5.4 statistical software for analysis. The risk of bias in randomized controlled trials was classified as low, high, or uncertain with regard to selection bias, performance bias, detection bias, attrition bias, reporting bias, and other biases. Reviewers (J.b.A.K. and T.F.T.K.) discussed and resolved any disagreement between the two previous reviewers (R.L. and K.Y.).

### Certainty of Evidence

The certainty of the evidence was evaluated for each meta-analysis utilizing the Grading of Recommendations, Assessment, Development, and Evaluation (GRADE) approach (Atkins et al., 2004). The quality of evidence for each outcome was rated by two reviewers (R.L. and K.Y.). Before assigning ratings, each outcome's initial quality was deemed high. The rating of each outcome was downgraded by one level to moderate, low or very low, based on specific criteria: (a) items at risk of bias were assessed as high; (b) inconsistency: there was statistically significant heterogeneity, with an I^2^ value greater than 75%; (c) indirectness: there were differences in the population, intervention, outcome measures, and comparators; (d) imprecision: the optimal information size was less than 400 participants or the confidence intervals were with wide ranges; (e) there was a high risk of publication bias.

### Statistical Analysis

The data collected in this study were organized using a Microsoft Excel spreadsheet, with RevMan 5.4 software employed for data statistics and meta-analysis. The mean and standard deviation (SD) on LEMS metrics of pre- and post-intervention between the BFRT and control groups were utilized for calculating Hedge's g effect size (ES). ES was reported along with the 95% confidence interval (CI), categorized as follows for interpretation clarity: trivial (< 0.2), small (0.2–0.6), moderate (0.6–1.2), large (1.2–2.0), very large (2.0–4.0), and extremely large (> 4.0) (Hopkins et al., 2009). A random effects model was applied for meta-analysis to interpret the pooled effect size of BFRT (Borenstein et al., 2021). Furthermore, heterogeneity between studies was assessed with I^2^, with < 25% classified as low, 25% to 75% as moderate, and > 75% as high (Higgins et al., 2003). The Egger’s test was used to evaluate publication bias, and sensitivity analysis was conducted when the results of the Egger’s test were statistically significant (Sterne et al., 2008). The *p*-value of < 0.05 was defined as statistically significant.

### Subgroup Analyses

The subgroup analysis was conducted using a random effects model to assess the potential factors contributing to the heterogeneity that influenced the training effects of BFRT. The analysis focused on moderator factors of BFRT a priori, which included the type of exercise performed during BFRT combined with resistance training (BFR-RT) versus BFRT combined with sport-specific training (BFR-NRT), training frequency of BFRT (< 3 vs. ≥ 3 times/week), training duration (≤ 6 vs. > 6 weeks), arterial occlusion pressure (< 160 mmHg vs. ≥ 160 mmHg), and cuff width (≤ 7 cm vs. > 7 cm). The median split method was used to divide these moderator variables (Moran et al., 2018).

## Results

### Study Selection

**Figure 1:**
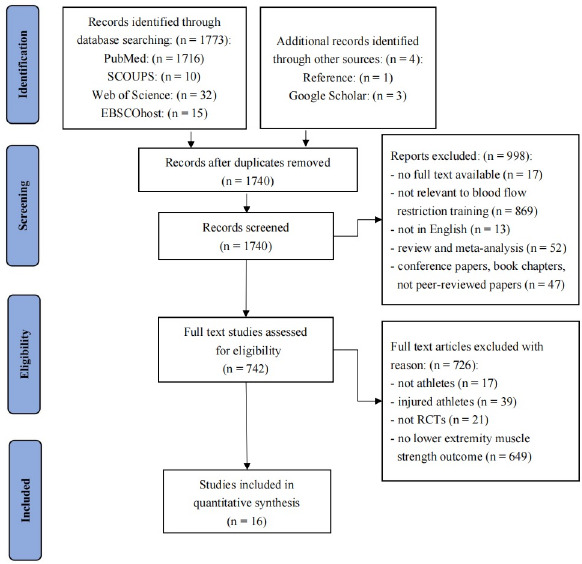
PRISMA flow diagram.

Figure 1 describes the search and screening process. A total of 1773 relevant publications were found through the electronic database search, and additional four papers were found through reference lists and Google Scholar. Among these studies, 1740 publications remained after manually deleting 37 duplicate documents. Furthermore, the titles and abstracts of these records were screened, and 742 articles were considered potentially valuable studies and their eligibility were assessed. Finally, 16 full-text articles satisfied the criteria and were included in the meta-analysis.

### Methodological Quality

**Figure 2:**
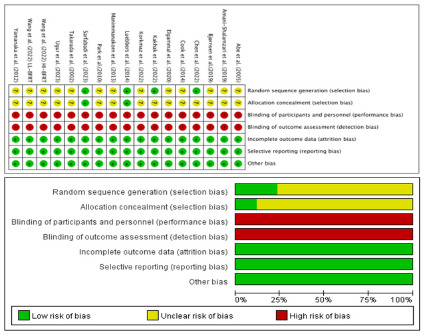
Risk of bias graph.

Figure 2 presents the results from the risk of bias assessment. Only four studies were at low risk of selection bias owing to the use of randomization procedures (Chen et al., 2022; Kakhak et al., 2022; Luebbers et al., 2014; Sarfabadi et al., 2023), and the remaining articles did not describe the detailed process, which was classified as having unclear bias. Notably, no study provided details on the assessment of blinding outcomes, all studies assigned a high-risk bias as the characterization of BFRT corresponded to the supervised exercise therapy study, which could not have blinded the investigators and participants. Furthermore, high dropout rates significantly contributed to the absence of missing data or less detailed records on missing data in 16 studies that were considered to have a low risk of attrition bias. The entire process reported the measurements; therefore, the reporting bias was of low risk.

### Certainty of Evidence

The three outcomes of the systematic review were evaluated using the GRADE approach, and the GRADE results suggested the low certainty of the evidence for the outcomes (Table 2).

**Table 2:** GRADE assessment of the results.

Outcomes	Certainty assessment					Certainty of Evidence (GRADE)
	Risk of bias	Inconsistency	Indirectness	Imprecision	Publication bias	
Lower extremity dynamic strength-1RM Follow-up: 1–8 weeks 200 (10 RCTs)	Serious^a^	Not serious	Not serious	Serious^b^	Not serious	⨁⨁◯◯ Low
Isokinetic knee -up: 3–8 weeks 190 (11 RCTs)	Serious^a^	Not serious	Not serious	Serious^b^	Not serious	⨁⨁◯◯ Low
Isokinetic Knee flexion peak torque Follow-up: 3–8 weeks 141 (8 RCTs)	Serious^a^	Not serious	Not serious	Serious^b^	Not serious	⨁⨁◯◯ Low

Note: RCTs = randomized controlled trials, GRADE = grading of recommendations assessment, development and evaluation, 1RM = one repetition maximum, ^a^ = downgraded one level by lack of blinding methods, ^b^ = downgraded one level due to optimal information size < 400.

### Study Characteristics

Sixteen articles published up to August 2024 included 366 athletes (Table 3). All studies reported on the type of the athlete, including rugby players (n = 2), sprinters (n = 1), soccer players (n = 4), basketball players (n = 2), netball players (n = 1), volleyball players (n = 1), endurance athletes (n = 1), fustal players (n = 1), powerlifters (n = 1), canoe athletes (n = 1), and long jumpers (n = 1). These athletes were between the ages of 15 and 27. Thirteen studies included male, and only one included female participants; two studies reported both males and females and one study did not provide information on gender. Furthermore, eleven studies conducted BFR-RT, most studies performed bilateral exercises, and only one study performed unilateral exercise (Korkmaz et al., 2022). Five studies performed BFR-NRT, including BFRT combined with walking, repeated sprint or small-side games. Moreover, the duration of BFRT ranged from two to eight weeks, with training frequency being two to six times per week. The training load was 20%–80% 1RM. The mode of BFRT in the most studies was continuous pressure, except for intermittent compression in only three studies (Amani-Shalamzari et al., 2019; Bjørnsen et al., 2019; Cook et al., 2014). The arterial occlusion pressures ranged between 100 and 240 mmHg, and only two studies used band tightness adjustment to represent pressure, by pulling the banding to overlap by 2 inches (Yamanaka et al., 2012) and 3 inches (Luebbers et al., 2014). The BFRT method employed in the included studies used pneumatic cuffs (n = 13) and elastic bands (n = 3). Of these, the range of cuff widths was 3.3 cm to 14.2 cm, with only three studies not reporting cuff width (Abe et al., 2005; Elgammal et al., 2020; Ugur et al., 2023).

**Table 3a:** Characteristics of studies included in the present review.

Studies	Population ( type of athletes, N, sex, age)	Training frequency/ length	Training protocol	BFR mode, cuff type, pressure, width	Comparison	Outcomes
Takarada et al., 2002	Elite rugby players, N = 12, M, EG: 25.3 ± 0.8 yr., CG: 26.5 ± 0.7 yr.	2 times/week, 8 weeks	Bilateral knee extension, 4 sets × 15–17 reps, 50% 1RM	Continuous, pneumatic cuffs, 200 mmHg, 3.3 cm	EG: BFR-RT, CG: NT	KEPT (Nm) ↑
Abe et al., 2005	University sprinters, N = 15, M, NR	2 times/day, 8 days	Bilateral squat and leg curl, 3 sets ×15 reps, 20% 1RM	Continuous, pneumatic cuffs, 160–240 mmHg, NR	EG: BFR-RT, CG: NT	Dynamic leg press 1RM ↑
Park et al., 2010	University basketball players, N = 12, M, EG: 20.1 ± 1.2 yr., CG: 20.8 ± 1.3 yr.	6 times/week, 2 weeks	5 sets × 3 min	Continuous, pneumatic cuffs, 160–220 mmHg, 11 cm	EG: BFRT with WT, CG: WT	KEPT (Nm) ↔, KFPT (Nm) ↑
Yamanaka et al., 2012	University soccer players, N = 32, M, 19.2 ± 1.8 yr.	3times/week, 4 weeks	Bilateral squat,1 set × 30 reps + 3 sets × 20 reps, 20% 1RM	Continuous, elastic bands, pulled to overlap 2 inches, 5 cm	EG: BFR-RT, CG: RT	Squat-1RM↑
Manimmanakorn et al., 2013	University netball players, N = 30, F, 20.2 ± 3.3 yr.	3 times/week, 5 weeks	Bilateral knee extension and flexion, 6 sets × 22–36 reps, 20% 1RM	Continuous, pneumatic cuffs, 160–230 mmHg, 5 cm	EG: BFR-RT, CG: RT	KEPT (Nm) ↑
Cook et al., 2014	Semiprofessional rugby players, N = 20, M, EG: 21.8 ± 1.2 yr., CG: 21.1 ± 1.5 yr.	3 times/week, 3 weeks	Bilateral squat, 5 sets × 5 reps, 70% 1RM	Intermittent, pneumatic cuffs, 180 mmHg, 10.5 cm	EG: BFR-RT, CG: RT	Squat-1RM↑
Luebbers et al., 2014	University soccer, players, N = 62, M, 20.3 ± 1.1 yr.	4 times/week, 7 weeks	Bilateral squat, 1 set × 30 reps + 3 sets × 20 reps, 20% 1RM	Continuous, elastic wraps, pulled to overlap 3 inches, 7.6 cm	EG: BFR-RT, CG: RT	Squat-1RM↑
Amani-Shalamzari et al., 2019	Elite fustal players, N = 12, M, 23 ± 2 yr.	3 times/week, 3 weeks	3min × 4–8 reps	Intermittent, pneumatic cuffs, 110–140 mmHg, 13 cm	EG: BFRT with SSG, CG: SSG	KEPT (Nm) ↑, KFPT (Nm) ↑
Bjørnsen et al., 2019	Elite powerlifters, N = 17, Mixed, EG: 24 ± 3 yr., CG: 26 ± 8 yr.	5 times/week, 6.5 weeks	Bilateral squat and deadlift, 4 sets × voluntary failure, 30% 1RM	Intermittent, elastic bands, 120 mmHg, 7.6 cm	EG: BFR-RT, CG: RT	KEPT (Nm) ↑, Squat-1RM↑
Elgammal et al., 2020	University basketball players, N = 24, M, 22.3 ± 2.4 yr.	3 times/week, 4 weeks	3 sets × 8 reps	Continuous, pneumatic cuffs, 100–160 mmHg, NR	EG: BFRT with RST, CG: RST	Squat-1RM↑
Wang et al., 2022 (HI-BFRT)	University volleyball players, N = 12, Mixed, EG: 20.17 ± 7.5 yr., CG: 20.83 ± 1.47 yr.	3 times/week, 8 weeks	Bilateral squat, 4 sets × 8 reps, 70% 1RM	Continuous, pneumatic cuffs, 180 mmHg, 7 cm	EG: BFR-RT, CG: RT	Squat-1RM↑, KEPT (Nm) ↑, KFPT (Nm) ↑
Wang et al., 2022 (LI-BFRT)	University volleyball players, N = 12, Mixed, EG: 20.50 ± 1.38 yr., CG: 20.83 ± 1.47 yr.	3 times/week, 8 weeks	Bilateral squat, 1 set × 30 reps + 3 sets × 15 reps, 30% 1RM	Continuous, pneumatic cuffs, 180 mmHg, 7 cm	EG: BFR-RT, CG: RT	Squat-1RM↑, KEPT (Nm) ↑, KFPT (Nm) ↑
Chen et al., 2022	University endurance athletes, N = 20, M, EG: 21.5 ± 0.78 yr., CG: 21.6 ± 0.75 yr.	3 times/week, 8 weeks	5 × 3min / 50% HRR	Continuous, pneumatic cuffs, 160 mmHg, 14.2 cm	EG: BFRT with RST, CG: RST	KEPT (Nm) ↑, KFPT (Nm)↑

**Table 3b:** Characteristics of studies included in the present review.

Studies	Population (type of athletes, N, sex, age)	Training frequency/ length	Training protocol	BFR mode, cuff type, pressure, width	Comparison	Outcomes
Korkmaz et al., 2022	Professional soccer players, N = 23, M, EG: 18.36 ± 0.5 yr., CG: 18.42 ± 0.79 yr.	2 times/week, 6 weeks	Unilateral leg extension, 1 set × 30 reps + 3 sets × 15 reps, 30% 1RM	Continuous, pneumatic cuffs, 130–150 mmHg, 7 cm	EG: BFR-RT, CG: RT	KEPT (Nm) ↑, KFPT (Nm) ↑
Kakhak et al., 2022	Adolescent soccer players, N = 19, M, 15.9 ± 0.8 yr.	3 times/week, 6 weeks	8–20 min	Continuous, pneumatic cuffs, 160–210 mmHg, 4 cm	EG: BFRT with SSG, CG: NT	Knee extension 1RM ↑
Sarfabadi et al., 2023	Long jumpers, N = 17, NR, NR	2 times/week, 6 weeks	Bilateral leg press and squat, 1 set × 30 reps + 3 sets × 15 reps, 20% 1RM	Continuous, pneumatic cuffs, 150 mmHg, 10 cm	EG: BFR-RT, CG: NT	Squat-1RM↑, KEPT (Nm) ↑, KFPT (Nm) ↑
Ugur et al., 2023	Elite canoe athletes, N = 33, M, EG: 18.59 ± 0.71 yr., CG: 18.81 ± 1.11 yr.	2 times/week, 8 weeks	Bilateral leg press, leg curl, and leg extension, 3 sets × 10–15 reps, 30% 1RM	Continuous, pneumatic cuffs, 180–230 mmHg, NR	EG: BFR-RT, CG: NT	KEPT (Nm) ↑, KFPT (Nm) ↑

Note: N = number, EG = experimental group, CG = control group, yr. = years, M = male, F = female, 1RM = one repetition maximum, BFRT = blood flow restriction training, BFR-RT = blood flow restriction combined with resistance training, RT = resistance training, NT = normal training, WT = walking training, RST = repeated sprint training, SSG = small side game training, KEPT = knee extension peak torque, KFPT = knee flexion peak torque, HRR = heart rate reserve, Reps = repetitions, Nm = newton meter, NR = not reported

### Meta-Analysis Results

#### Lower Extremity Dynamic Strength-1RM

**Figure 3:**
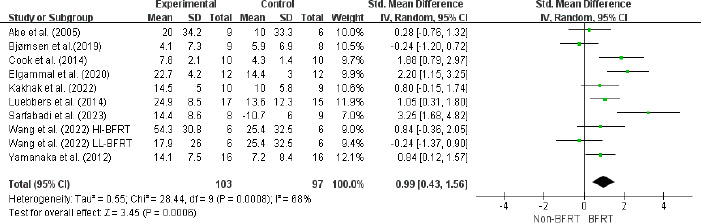
Forest plot for the effects of BFRT on lower extremity dynamic strength-1RM in athletes. Squares represent effect size (Hedges’ g) corresponding to weight of the study and lines are the 95% confidence interval (CI); diamond indicates overall Hedges’ g with its width corresponding to the 95% CI; BFRT = blood flow restriction training, Non-BFRT = control training

A meta-analysis of nine studies involving 224 athletes in ten experimental groups demonstrated that BFRT significantly enhanced lower extremity dynamic strength-1RM (ES = 0.99; 95% CI = [0.43–1.56]; Z = 3.45; *p* = 0.0006; I^2^ = 68%; Egger’s test: *p* = 0.380; Figure 3).

#### Isokinetic Knee Extension Peak Torque

**Figure 4:**
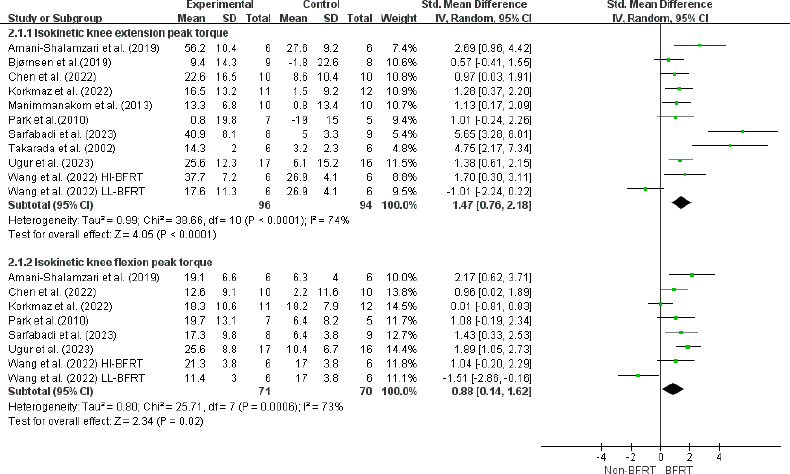
Forest plot for the effects of BFRT on isokinetic muscle strength in athletes. Squares represent effect size (Hedges’ g) corresponding to weight of the study and lines are the 95% confidence interval (CI); diamond indicates overall Hedges’ g with its width corresponding to the 95% CI; BFRT = blood flow restriction training, Non-BFRT = control training

The isokinetic knee extension peak torque was assessed in ten studies with 190 athletes. There was a significant improvement after BFRT (ES = 1.47; 95% CI = [0.76–2.18]; Z = 4.05; *p* < 0.001; I^2^ = 74%; Egger’s test: *p* = 0.917; Figure 4). The results of the subgroup analysis indicated that BFR-RT (ES = 1.95; 95% CI = [1.05–2.85]; *p* < 0.001) produced more considerable effects. It was more beneficial to perform BFRT ≥ 3 times per week (ES = 1.13; 95% CI = [0.66–1.59]; *p* < 0.001) than < 3 times per week (ES = 0.80; 95% CI = [0.17–1.25]; *p* = 0.10). Similarly, the occlusion pressure of ≥ 160 mmHg (ES = 2.23; 95% CI = [0.63–3.83]; *p* = 0.006) and the cuff width of BFRT > 7 cm (ES = 1.84; 95% CI = [0.59–3.10]; *p* = 0.004) were significantly increased. Moreover, no significance was observed in the duration of BFRT (Table 4).

**Table 4:** Effects of moderating variables on isokinetic muscle strength in BFRT.

Subgroup	Trials	Effect size and confidence interval	*p*	Within groupI^2^ (%)	Within group*p*	Between group I^2^ (%)	Between group*p*
Isokinetic knee extension peak torque							
BFR-RT	5	1.95 (1.05, 2.85)	< 0.001	74	0.0008	0	0.35
BFR-NRT	3	1.35 (0.44, 0.25)	0.004	36	0.21		
< 3 times/ week	4	0.80 (0.17, 1.25)	0.10	48	0.03	60.6	0.03
≥ 3 times/ week	6	1.13 (0.66-1.59)	< 0.001	3	0.40		
≤ 6 weeks	5	1.99 (0.86, 3.11)	< 0.001	0	0.37	0	0.58
> 6 weeks	5	1.54 (0.37, 2.70)	0.010	69	0.005		
< 160 mmHg	4	1.53 (0.88, 2.19)	0.09	47	0.09	0	0.43
≥ 160 mmHg	6	2.23 (0.63, 3.83)	0.006	70	0.0006		
≤ 7 cm	5	1.28 (0.03, 2.53)	0.05	68	0.006	0	0.53
> 7 cm	5	1.84 (0.59, 3.10)	0.004	57	0.001		
Isokinetic knee flexion peak torque							
BFR-RT	4	1.20 (0.52, 1.87)	0.02	0	0.40	0	0.84
BFR-NRT	3	0.42 (−0.57, 1.41)	0.41	46	0.01		
< 3 times/ week	3	1.09 (−0.11, 2.30)	0.07	59	0.005	0	0.62
≥ 3 times/ week	4	1.16 (0.57, 1.73)	< 0.001	0	0.59		
≤ 6 weeks	4	0.92 (−0.29, 2.13)	0.14	58	0.05	0	0.55
> 6 weeks	3	1.37 (0.74, 2.00)	< 0.001	19	0.29		
< 160 mmHg	3	1.08 (−0.20, 2.37)	0.10	74	0.02	0	0.73
≥ 160 mmHg	4	1.32 (0.81, 1.83)	< 0.001	0	0.43		
≤ 7 cm	3	−0.49 (−7.38, 6.39)	0.89	82	0.003	72.2	0.002
> 7 cm	4	11.84 (7.89, 15.79)	< 0.001	0	0.96		

Note: BFR-RT = blood flow restriction training combined with resistance training, BFR-NRT = blood flow restriction training combined with sport-specific training.

#### Isokinetic Knee Flexion Peak Torque

The pooled results from seven studies including 141 athletes indicated a moderate effect size in isokinetic flexion after BFRT (ES = 0.88; 95% CI = [0.14–1.62]; Z = 2.34; *p* = 0.02; I^2^ = 73%; Egger’s test: *p* = 0.054; Figure 4). Subgroup analysis demonstrated that BFR-RT training programs (ES = 1.20; 95% CI = [0.52–1.87]; *p* = 0.02) performed ≥ 3 times per week (ES = 1.16; 95% CI = [0.57–1.73]; *p* < 0.001) with occlusion cuff pressures of ≥ 160 mmHg (ES = 1.32; 95% CI = [0.81–1.83]; *p* < 0.001) and the cuff width of > 7 cm (ES = 11.84; 95% CI = [7.89–15.79]; *p* < 0.001) were more effective in increasing lower extremity strength (Table 4).

## Discussion

This study is the first systematic review and meta-analysis to examine the impact of BFRT on LEMS in healthy athletes. The results revealed that BFRT provided moderate to large increases (ES = 0.88–1.47) in athletes' lower extremity dynamic strength-1RM, isokinetic knee extension peak torque, and isokinetic knee flexion peak torque. These findings exhibited moderate heterogeneity, and the GRADE analysis indicated a low certainty of evidence. Additionally, the present study conducted subgroup analyses to investigate moderators of isokinetic muscle strength, with each moderator variable including at least three studies. The findings suggested that BFRT combined with RT, training frequency of ≥ 3 times per week, and occlusion pressure of ≥ 160 mmHg significantly improved isokinetic muscle strength in athletes. Conversely, no significant moderating effect was observed for the training duration of BFRT.

1RM serves as a benchmark to assess maximal dynamic muscular strength, representing the maximum force a subject can exert during the complete execution of a specific movement (Verdijk et al., 2009). The results showed the effectiveness of BFRT in enhancing squat-1RM in athletes (ES = 0.99). These findings align with those of a previous systematic review which demonstrated the beneficial impact of BFRT on dynamic strength-1RM in athletes (Wortman et al., 2021). The present study corroborates and reinforces these findings through meta-analysis. Several studies have reported the benefits of BFRT on the dynamic strength of lower extremities across diverse populations (Gujral et al., 2023; Lixandrão et al., 2018; Rodrigues et al., 2020; Vechin et al., 2015; Wang et al., 2022). The enhancement in dynamic muscle strength following BFRT may be associated with the theory proposed by Sale (1988) who posited that a blend of neural adaptations and muscle hypertrophy contribute to increases in strength. The metabolic accumulation occurring under blood flow-restricted conditions inhibits alpha motor neuron excitation, thereby modifying motor unit (MU) recruitment patterns (Yasuda et al., 2010). MU augmentation occurs after cuff pressure release, which amplifies force output and induces muscle hypertrophy (Centner et al., 2019). Furthermore, one study that examined muscles after BFRT combined with RT revealed that the BFRT group exhibited a higher percentage of type II fibers than a control group without occlusion training (Krustrup et al., 2009). These findings substantiate the blood flow restriction training can facilitate heightened activation of muscle fiber types and the MU threshold, resulting in improved dynamic muscle strength of lower limb.

Lower extremity isokinetic muscle strength plays a crucial role in enabling athletes to control movement rhythms and maintain stability, which represents the force exerted to maintain a consistent velocity during muscle contraction and extension (Gaines and Talbot, 1999). The joint motion requires muscle contraction to achieve proper flexion and extension, which can stimulate muscle fibers to recruit additional muscle units to enhance strength (Wretenberg et al., 1993). Generally, the peak torque serves as the metric to evaluate the degree of muscle contraction throughout the range of joint movement (Montgomery et al., 1989). Our findings align with previous meta-analyses (Yang et al., 2022), whereas the present study specifically targeted athletes. Slysz et al. (2016) explored the positive effects of BFRT on isokinetic knee extension and flexion strength can be explained by neuromuscular activation. The activation of the post-activation potentiation (PAP) effect is considered a potential mechanism for enhancing muscle adaptation (Hodgson et al., 2005). This effect could elevate the sensitivity of neurons to external stimuli, resulting in heightened excitability levels within the central nervous system under the vascular occlusion environment, improving muscle activation and force output (Yasuda et al., 2009). However, a meta-analysis revealed that long-term RT without occlusion did not appear to enhance neuromuscular excitability (Siddique et al., 2020). Interestingly, Park et al. (2010) found that two weeks of BFRT combined with HI-RT had no significant effect on knee extension peak torque in basketball players. This discrepancy could be attributed to inadequate duration of training, leading to insufficient neuromuscular adaptation (Folland and Williams, 2007). Therefore, the relative contribution of BFRT to neuromuscular activation may be varied by the duration of BFRT, and future research could explore this aspect further in athletes.

The results indicated that the type of training, frequency, and occlusion pressure of BFRT had mediating effects. BFR-RT appears to be more advantageous than BFRT combined with sport-specific training for enhancing LEMS in athletes, which may be attributed to the exercises specific for the sport (Gamble, 2006). Additionally, the findings demonstrated that BFRT was more effective with frequency of ≥ 3 sessions per week. This is in line with Yang et al. (2022) who indicated in their meta-analysis that training frequency of > 3 times per week showed the best effects of BFRT on LEMS. High-frequency exercise is associated with a frequent recovery process, aiding in muscle protein synthesis and muscle growth promotion, thus the effects of BFRT gradually appear as the training frequency increases (Chang et al., 2022). However, Pavlou et al. (2023) suggested that BFRT with training frequency of 2–3 times per week produced good muscle adaptation in the upper limb. This slight difference could be explained as the cuffs in the upper limb are applied proximal to the muscles, whereas the muscle groups in the lower limb are mainly located distally to the application of the occlusive pressure (Dankel et al., 2016). Furthermore, we observed that a limb occlusion pressure ≥ 160 mmHg during BFRT may be more beneficial. Higher occlusion pressure can result in the accumulation of lactate metabolites and notable changes in venous oxygen saturation and growth hormone secretion (Fatela et al., 2018). Indeed, the meta-analysis by Loenneke et al. (2012) was unable to analyse the overall effect of cuff width on the effect of BFRT as the cuff widths of the included studies were less than 5 cm. Nevertheless, our subgroup analyses found that BFRT with wider cuffs of > 7 cm led to a more significant enhancement in athletes' LEMS. This finding is supported by Rossow et al. (2012) who stated that a 13.5-cm cuff increased the distance over which pressure was applied to the tissues to a greater extent than a 5-cm cuff, producing greater resistance to the blood flow. Conversely, Jessee et al. (2016) suggested that narrower cuffs could be more effective in promoting muscle growth. This may be related to differences in limb circumferences, with larger limbs requiring higher pressures to stimulate deeper muscle tissue. Furthermore, one study indicated that different widths appeared to evoke similar blood flow responses when using the same arterial occlusion pressure (Mouser et al., 2017). The effect of cuff width on the effectiveness of BFRT may not be solely dependent on the width itself, and it is recommended that future studies investigate the relationship between cuff width and the applied pressure.

Several limitations of this review are notable. First, most of the studies included in this review were not registered online and had unclear randomization, which means that the risk of selective reporting bias could not be fully addressed, potentially decreasing the credibility of the articles. Second, most of the included publications did not clearly specify occlusion pressure values and only provided pressures within a specific range (i.e., 160–220 mmHg). Therefore, future studies should aim to determine optimal blood restriction pressure and explore BFRT in combination with specific training to strengthen the validity of findings and provide additional theoretical support for BFRT.

## Conclusions

This meta-analysis suggests that BFRT can effectively improve lower extremity maximum dynamic strength and isokinetic muscle strength in healthy athletes. Furthermore, subgroup analysis indicates that combining BFRT with traditional RT appears to be more effective in enhancing muscle strength than combining BFRT with sport-specific exercises. BFRT sessions conducted at a frequency of ≥ 3 times per week, limb occlusion pressure of ≥ 160 mmHg and cuff width of > 7 cm significantly enhance lower limb muscle strength in athletes. Notably, contradictory findings regarding the moderating effects of training duration on BFRT were identified, emphasizing the need for further studies to determine the effectiveness of specific training combined with BFRT among athletes.
